# Supplementary material for: Gut microbiome compositional clusters in association with cardiovascular risk: An observational cohort study
Source: PLoS One. 2026 Feb 6;21(2):e0341111. doi: 10.1371/journal.pone.0341111 (PMC12880714; doi:10.1371/journal.pone.0341111)
Supplement: S1 Table — (DOCX) [file pone.0341111.s001.docx]

|  | **Hazard ratio** | **95% Confidence interval** | **P-value** |
| --- | --- | --- | --- |
| **Main Multivariable Model** |  |  |  |
| Clusters (L compared to H) | 0.48 | 0.26 – 0.91 | 0.024 |
| Age, years | 1.07 | 1.03 – 1.11 | 0.001 |
| Body mass index, Kg/m^2^ | 1.03 | 0.98 – 1.08 | 0.231 |
| Coronary artery disease | 2.21 | 1.06 – 4.60 | 0.034 |
| Hyperlipidemia | 1.98 | 0..66 – 5.95 | 0.225 |
| Hypertension | 1.20 | 0.51 – 2.84 | 0.675 |
| **Interaction test** |  |  |  |
| Clusters (L compared to H) | 12.23 | 0.04 - 395 | 0.396 |
| Age, years | 1.19 | 1.09 – 1.31 | <0.001 |
| Coronary artery disease | 3883 | 5.87 - 2570968 | 0.013 |
| Clusters * Age | 0.94 | 0.87 – 1.01 | 0.101 |
| Clusters * coronary artery disease | 4.11 | 0.70 – 24.08 | 0.117 |
| Age * coronary artery disease | 0.89 | 0.81 – 0.98 | 0.016 |
| **Demographics Multivariable Model** |  |  |  |
| Clusters (L compared to H) | 0.48 | 0.25 - 0.91 | 0.025 |
| Age, years | 1.08 | 1.04 - 1.12 | <0.001 |
| Body mass index, Kg/m^2^ | 1.04 | 0.99 - 1.09 | 0.11 |
| Male sex | 1.26 | 0.65 - 2.42 | 0.5 |
| Physical exercise | 0.79 | 0.42 - 1.46 | 0.4 |
| Low fat diet | 1.16 | 0.60 - 2.27 | 0.7 |
| **Comorbidities Multivariable Model** |  |  |  |
| Clusters (L compared to H) | 0.49 | 0.26 - 0.92 | **0.026** |
| Coronary artery disease | 2.94 | 1.36 - 6.36 | **0.006** |
| Hyperlipidemia | 2.33 | 0.75 - 7.20 | 0.14 |
| Hypertension | 1.76 | 0.73 - 4.26 | 0.2 |
| Diabetes mellitus | 1.17 | 0.61 - 2.21 | 0.6 |
| **Medications Multivariable Model** |  |  |  |
| Clusters (L compared to H) | 0.54 | 0.28 - 1.02 | 0.059 |
| Lipid lowering medications | 2.14 | 0.99 - 4.64 | 0.053 |
| Antihypertensive | 2.20 | 0.70 - 6.94 | 0.2 |
| Proton pump inhibitors | 1.77 | 0.94 - 3.33 | 0.075 |
| Beta-blockers | 1.88 | 0.84 - 4.21 | 0.13 |
| Antiplatelets | 1.06 | 0.43 - 2.64 | 0.9 |
