# Supplementary material for: Gut microbiome compositional clusters in association with cardiovascular risk: An observational cohort study
Source: PLoS One. 2026 Feb 6;21(2):e0341111. doi: 10.1371/journal.pone.0341111 (PMC12880714; doi:10.1371/journal.pone.0341111)
Supplement: S2 Table — (DOCX) [file pone.0341111.s002.docx]

|  | **Total Cohort**  **(N=211)** | | | | **CAD (+)**  **(N=92)** | | | | **CAD (-)**  **(N=119)** | | | |
| --- | --- | --- | --- | --- | --- | --- | --- | --- | --- | --- | --- | --- |
|  | **DF** | **R^2^** | **F** | **P-value** | **DF** | **R^2^** | **F** | **P-value** | **DF** | **R^2^** | **F** | **P-value** |
| **Age** | 1 | 0.025 | 5.639 | <0.001 | 1 | 0.013 | 1.210 | 0.207 | 1 | 0.034 | 4.242 | <0.001 |
| **Body Mass Index** | 1 | 0.023 | 5.110 | <0.001 | 1 | 0.023 | 2.126 | 0.006 | 1 | 0.030 | 3.840 | <0.001 |
| **Gender** | 1 | 0.007 | 1.611 | 0.056 | 1 | 0.012 | 1.087 | 0.297 | 1 | 0.015 | 1.907 | 0.015 |
| **Exercise** | 1 | 0.008 | 1.815 | 0.039 | 1 | 0.009 | 0.877 | 0.579 | 1 | 0.014 | 1.818 | 0.028 |
| **Alcohol** | 1 | 0.006 | 1.261 | 0.184 | 1 | 0.014 | 1.352 | 0.134 | 1 | 0.011 | 1.361 | 0.154 |
| **Current smoking** | 1 | 0.005 | 1.049 | 0.369 | 1 | 0.008 | 0.713 | 0.787 | 1 | 0.008 | 1.051 | 0.334 |
| **Low-fat diet** | 1 | 0.004 | 0.935 | 0.483 | 1 | 0.015 | 1.416 | 0.110 | 1 | 0.006 | 0.704 | 0.840 |
| **Hypertension** | 1 | 0.005 | 1.201 | 0.219 | 1 | 0.010 | 0.953 | 0.500 | 1 | 0.007 | 0.915 | 0.554 |
| **Diabetes mellitus** | 1 | 0.002 | 0.448 | 0.984 | 1 | 0.007 | 0.708 | 0.808 | 1 | 0.005 | 0.692 | 0.795 |
| **Coronary artery disease** | 1 | 0.005 | 1.180 | 0.244 | - | - | - | - | - | - | - | - |
| **Antihypertensive medications** | 1 | 0.006 | 1.316 | 0.166 | 1 | 0.011 | 1.063 | 0.346 | 1 | 0.006 | 0.763 | 0.727 |
| **Antidiabetic medications** | 1 | 0.010 | 2.250 | 0.009 | 1 | 0.0142 | 1.342 | 0.136 | 1 | 0.017 | 2.085 | 0.014 |
| **Lipid-lowering medications** | 1 | 0.004 | 0.882 | 0.565 | 1 | 0.016 | 1.532 | 0.079 | 1 | 0.004 | 0.524 | 0.962 |
| **Beta-blockers** | 1 | 0.011 | 2.557 | 0.003 | 1 | 0.021 | 1.965 | 0.016 | 1 | 0.009 | 1.175 | 0.258 |
| **Proton pump inhibitors** | 1 | 0.008 | 1.745 | 0.029 | 1 | 0.012 | 1.150 | 0.285 | 1 | 0.012 | 1.521 | 0.083 |
| **Residual** | 195 | - | 0.871 | - | 77 | 0.815 | - | - | 104 | 0.821 | - | - |
| **Total** | 210 | - | 1 | - | 91 | 1 | - | - | 118 | 1 | - | - |
