# Supplementary material for: Gut microbiome compositional clusters in association with cardiovascular risk: An observational cohort study
Source: PLoS One. 2026 Feb 6;21(2):e0341111. doi: 10.1371/journal.pone.0341111 (PMC12880714; doi:10.1371/journal.pone.0341111)
Supplement: S3 Table — (DOCX) [file pone.0341111.s003.docx]

| **feature** | **metadata** | **value** | **coef** | **stderr** | **N** | **N.not.0** | **pval** | **qval** |
| --- | --- | --- | --- | --- | --- | --- | --- | --- |
| Bacteroidetes.Bacteroidia.Bacteroides.CN | Cluster | L | 0.167157 | 0.008074 | 211 | 211 | 1.25E-51 | 3.01E-48 |
| Bacteroidetes.Bacteroidia.Bacteroides.CM | Cluster | L | 0.001184 | 0.000131 | 211 | 200 | 1.22E-16 | 1.46E-13 |
| Bacteroidetes.Bacteroidia.Bacteroides.DQ | Cluster | L | 0.036008 | 0.004256 | 211 | 211 | 5.62E-15 | 4.51E-12 |
| Bacteroidetes.Bacteroidia.Bacteroides.DP | AGE | AGE | -1.9E-06 | 3.68E-07 | 211 | 61 | 4.04E-07 | 0.000243 |
| Bacteroidetes.Bacteroidia.Bacteroides.DS | Cluster | L | 0.014742 | 0.00297 | 211 | 211 | 1.48E-06 | 0.000712 |
| Firmicutes.Clostridia.Clostridiales.LJ | AGE | AGE | -0.0006 | 0.000122 | 211 | 98 | 1.98E-06 | 0.000796 |
| Firmicutes.Clostridia.Clostridiales.LL | Cluster | L | -0.0523 | 0.011289 | 211 | 211 | 6.51E-06 | 0.00224 |
| Firmicutes.Clostridia.Clostridiales.LS | Cluster | L | -0.00917 | 0.002014 | 211 | 210 | 9.07E-06 | 0.002733 |
| Firmicutes.Clostridia.Clostridiales.LH | Cluster | L | -0.00806 | 0.00185 | 211 | 211 | 2.11E-05 | 0.005093 |
| Firmicutes.Clostridia.Clostridiales.OU | BMI | BMI | -0.00022 | 4.93E-05 | 211 | 192 | 1.97E-05 | 0.005093 |
| Firmicutes.Clostridia.Clostridiales.OF | Cluster | L | 0.002927 | 0.000679 | 211 | 211 | 2.55E-05 | 0.005584 |
| Actinobacteria.Actinobacteria.Microc.K | PPI | Yes | 2.63E-05 | 6.43E-06 | 211 | 42 | 6.43E-05 | 0.011926 |
| Firmicutes.Clostridia.Clostridiales.NY | Cluster | L | 0.002984 | 0.000728 | 211 | 204 | 6.09E-05 | 0.011926 |
| Proteobacteria.Alphaproteobacteria.S.D | BMI | BMI | 1.25E-06 | 3.1E-07 | 211 | 31 | 7.89E-05 | 0.013398 |
| Proteobacteria.Gammaproteobacteria.E.N | Antidiabetics | Yes | 0.053977 | 0.013437 | 211 | 204 | 8.34E-05 | 0.013398 |
| Firmicutes.Clostridia.Clostridiales.OP | BMI | BMI | -0.0001 | 2.58E-05 | 211 | 204 | 0.000107 | 0.016139 |
| Firmicutes.Clostridia.Clostridiales.NA | Diet | Strictly | 0.001386 | 0.000358 | 211 | 171 | 0.000144 | 0.020391 |
| Bacteroidetes.Bacteroidia.Flavobacte.I | Diabetes | Yes | 0.004273 | 0.001132 | 211 | 51 | 0.000211 | 0.02828 |
| Actinobacteria.Actinobacteria.Actino.L | Alcohol use | > 3 | 0.00034 | 9.21E-05 | 211 | 199 | 0.000285 | 0.03574 |
| Firmicutes.Clostridia.Clostridiales.KP | BMI | BMI | -3.5E-06 | 9.61E-07 | 211 | 122 | 0.000297 | 0.03574 |
| Actinobacteria.Actinobacteria.Actino.L | Cluster | L | -0.00026 | 7.28E-05 | 211 | 199 | 0.000401 | 0.046059 |
