# Supplementary figures and images for: Gut microbiome compositional clusters in association with cardiovascular risk: An observational cohort study
Source: PLoS One. 2026 Feb 6;21(2):e0341111. doi: 10.1371/journal.pone.0341111 (PMC12880714; doi:10.1371/journal.pone.0341111)

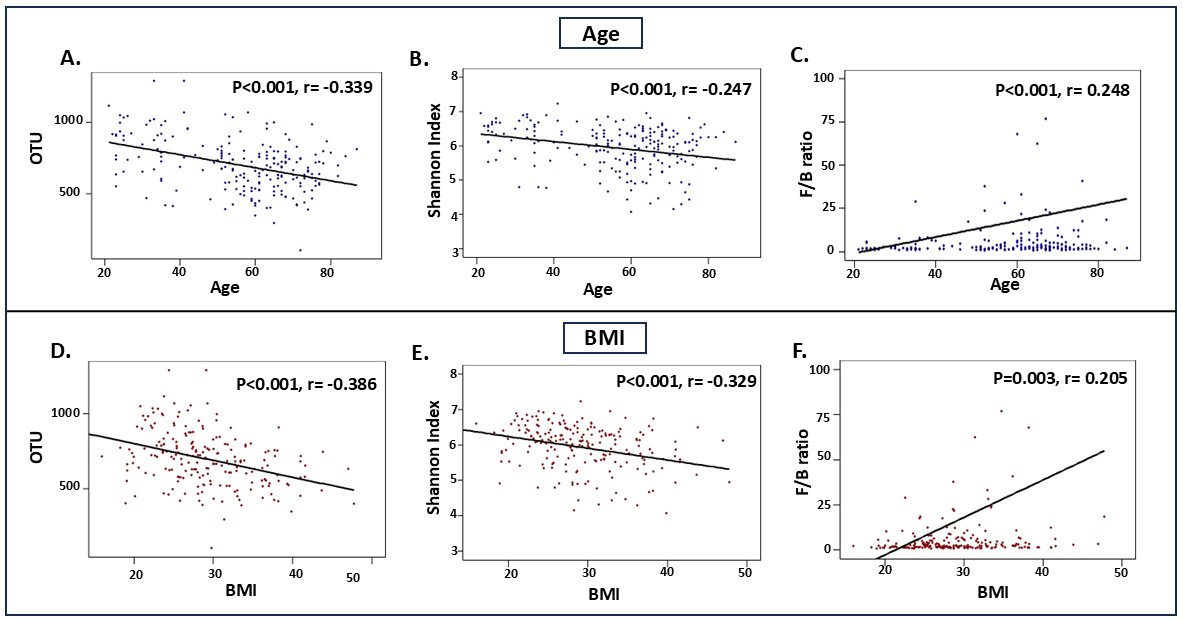

Supplement: S1 Fig — A. Scatterplot representing the correlation between age and OTUs. There was a moderate and inverse correlation between age and number of OTUs. B. Scatterplot illustrating the correlation between Shannon index and age. There was a gradual decrease in the Shannon index with advancing age. C. Scatterplot depicting the correlation between age and F/B ratio. There was a weak but statistically significant correlation between age and the F/B ratio. D. Scatterplot of the correlation between OTUs and BMI. There was a moderate negative correlation between number of OTUs and BMI. E. Scatterplot of the correlation between Shannon index and BMI. There was a moderate negative correlation between BMI and Shannon index. F. Scatterplot of the correlation between BMI and F/B ratio. There was a significant correlation between BMI and F/B ratio. BMI: Body Mass Index (Kg/m2), F/B ratio: Bacillota to Bacteroidetes ratio, OTU: Operational Taxonomic Units. (TIF) [file pone.0341111.s004.tif]

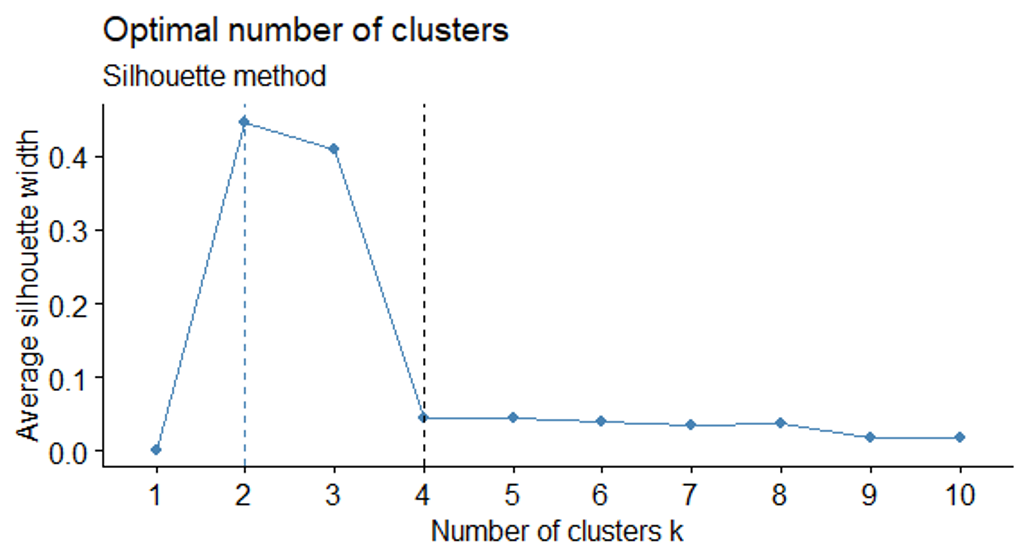

Supplement: S2 Fig — (TIF) [file pone.0341111.s005.tif]
